# Maternal morbidity associated with skin incision type at cesarean delivery in obese patients: a systematic review

**DOI:** 10.2144/fsoa-2020-0160

**Published:** 2020-12-18

**Authors:** Dani Zoorob, Oxana Zarudskaya, James Van Hook, Hind N Moussa

**Affiliations:** 1 Department of Obstetrics & Gynecology, University of Toledo College of Medicine & Life Sciences, Toledo, OH 43606, USA; 2 Division of Maternal-Fetal Medicine, The University of Cincinnati, Cincinnati, OH 45267, USA; 3 Kettering Physician Network Women’s Health – Kettering Maternal–Fetal Medicine, Kettering, OH 45429, USA

**Keywords:** cesarean delivery, obesity, transverse skin incision, vertical skin incision, wound complication

## Abstract

**Aim:**

To describe the relationship between cesarean skin incision type and postoperative wound complications (WCs) in obese pregnant patients.

**Materials & methods:**

MEDLINE (PubMed and OVID), Embase, Scopus, Web of Science Core Collection, Cochrane Library and ClinicalTrials.gov databases were used for publication search. Selection criteria consisted of articles studying pregnant patients with BMI ≥30 kg/m^2^ undergoing cesarean delivery and assessing the effect of skin incision type on postoperative maternal outcomes.

**Results:**

Ten publications met criteria for a systematic review of a total of 2946 patients. The transverse skin incision was associated with a lower rate of WC compared with the vertical skin incision. The pooled risk ratio for WCs was 0.47 (95% CI: 0.37–0.58; p < 0.00001).

**Conclusion:**

Transverse skin incision may be preferable to vertical skin incision at cesarean delivery in pregnant patients with obesity as it may be associated with a lower rate of WCs.

**PROSPERO registration ID:** CRD42020151106

## Lay abstract

This systematic review describes the relationship between skin incision type and postoperative wound complications in obese patients following cesarean delivery. The study results suggest that transverse skin incisions may be preferable to vertical skin incisions in the obese obstetric population for the reduction of wound complications.

Cesarean delivery (CD) is one of the most common abdominal surgeries worldwide, representing a third of deliveries in the USA and even higher rates among obese patients [1–3].

Obesity is particularly prevalent in today’s society. In the USA, 37% of women aged 20–39 years are obese (BMI ≥30 kg/m^2^), and 10.1% have class III obesity (BMI ≥40 kg/m^2^) [2–5]. Obesity during pregnancy increases the risk of failed induction, failed vaginal birth after primary cesarean section, gestational diabetes, macrosomia and cephalopelvic disproportion, all factors which increase the risk of CD [6–8].

Research has shown that the risk of undergoing a CD is twofold higher in women with a BMI >35 kg/m^2^ [3]. Other studies have also demonstrated that obesity increases the risk of wound complications (WCs) following CD, the risk of prolonged operative time as well as the rate of postpartum hemorrhage [9–14]. A study reviewing over 142,404 pregnancies reported a higher wound infection rate among obese compared with nonobese patients [15].

The aim of this study was to describe the relationship between cesarean skin incision type and postoperative WCs as well as other outcomes in obese women using the systematic review method.

## Materials & methods

We searched MEDLINE (PubMed and OVID), Embase, Scopus, Web of Science Core Collection, Cochrane Library and ClinicalTrials.gov databases for relevant studies evaluating the association between cesarean skin incision types and maternal morbidity in obese pregnant patients. Search terms included ‘cesarean section’, ‘abdominal or skin incision’, ‘vertical skin incision’, ‘Pfannenstiel’, ‘transverse skin incision’, ‘midline incision’, ‘supra-pannicular incision’, ‘obese’ and ‘body mass index’. Supplementary Appendix 1 illustrates the search strategy used for each database. The database searches were without restriction on study design type or date of publication. We further reviewed the reference section of the eligible articles to identify other relevant studies that may not have appeared in the literature search.

This systematic review was performed in accordance with version 5.1.0 of the Cochrane handbook for systematic reviews of interventions [16] and the PRISMA statement [17]. Two reviewers (O Zarudskaya and HN Moussa) independently screened the abstracts of all records identified through the initial search. Identified texts underwent inclusion analysis. We only included studies that compared the effectiveness of different skin incision types on postoperative maternal morbidity after CD in women with BMI ≥30 kg/m^2^. The primary outcomes addressed WCs, whereas secondary outcomes focused on other morbidities such as blood loss and transfusion rate, operative time, and duration of hospital stay. Studies were excluded if they had insufficient or duplicate data, did not report maternal outcomes, or performed on the nonobese population. Any discrepancy during the study selection process was resolved by discussion.

Extracted data included the last name of the first author, year of publication, country of origin, study design and study period. Additionally, study population information was included, such as the number of patients, maternal age, gestational age, BMI, type of cesarean incision, number of patients with preeclampsia, history of chronic hypertension or diabetes and tobacco use. Additionally, prophylactic antibiotics administration, classical hysterotomy, WC, estimated blood loss, blood transfusion, length of postoperative hospital stay and operative time were recorded. Data are presented in Table 3. The definition of WC was based on the respective study’s definition and preserved as such.

We evaluated the quality of the eligible cohort studies using the quality assessment tool of the Newcastle Ottawa scale [18]. The risk of bias in the randomized controlled trial (RCT) was assessed following the Cochrane risk of bias assessment tool; authors’ judgments for bias were segmented as ‘low risk’, ‘high risk’ or ‘unclear risk’ [19] (see Supplementary Appendix 2A & B).

WC and blood transfusion rates were summarized using percentages, with an adjusted odds ratio with a 95% CI, if available. Continuous data were reported as mean ± standard deviation or median (1st–3rd quartile). We pooled results when possible. Pooled estimates were obtained using the RevMan 5.3.5 (The Nordic Cochrane Centre, Copenhagen, Denmark) software for Windows. Heterogeneity was assessed by the I^2^ statistic and chi-square (X^2^) test. Fixed effects were used when no heterogeneity was detected. We did not assess funnel plot asymmetry for the existence of publication bias, since it is not reliable for <10 pooled studies, according to Egger *et al.* [20].

## Results

**Figure 1.**
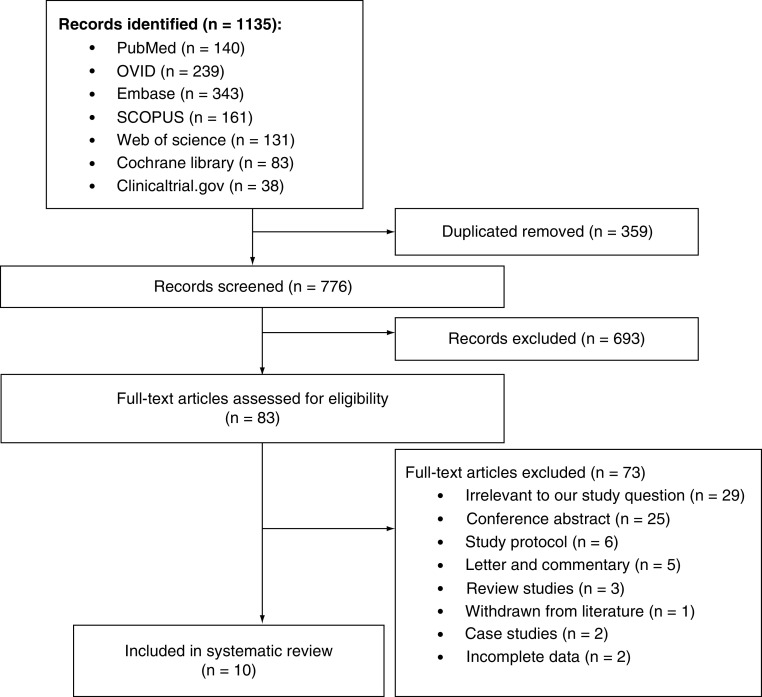
PRISMA flow diagram of literature search and study selection process.

Our search identified 1135 records. After 359 duplicates were removed, 776 titles and abstracts were screened to yield 83 studies for full-text assessment. Ten articles met our prespecified inclusion criteria, including a total of 2946 obese pregnant patients who underwent CD (Figure 1). Of these ten publications, nine were retrospective cohort studies, and one was a RCT. No further studies were retrieved from our manual search, which was accomplished through screening the reference sections of the final included articles and trials from ClinicalTrials.gov. Articles meeting review criteria were those published in journals as full-text articles from 2003 to 2019. All included cohort studies were at low risk of bias, with scores varying from 7 to 9 when the Newcastle-Ottawa scale was applied. The RCT (Marrs *et al.*) trial met a low risk of bias criteria when assessed using the Cochrane tool [27]. The study design, sample size, time of data collection, types of incision and the reported primary outcome for each study are presented in Table 1.

**Table 1.** Characteristics and validity of included studies.

Study (year), country	Study design	Dates of data collection	Inclusion criteria	Sample size, n	Types of incision		Primary outcome; definition	Ref.
Sutton *et al.* (2015), USA	Retrospective cohort	2010–2013	Primary or repeated CD GA >34 weeks BMI >40 kg/m^2^	423	Transverse, subpannicular	Vertical	WC; any of cellulitis, abscess, hematoma, seroma or fascial dehiscence	[22]
Bell *et al.* (2011), USA	Retrospective cohort	2004–2006	Primary or repeated CD BMI >35 kg/m^2^	424	Low transverse, irrespective of distance from the pubic bone superior border	Vertical, longitudinal incision, infra or supraumbilical	Wound infection and/or breakdown; wound separation and/or purulent discharge with or without cellulitis or fever	[21]
Alanis *et al.* (2010), USA	Retrospective cohort	2005–2009	Primary or repeated CD GA 20–44 weeks BMI ≥50 kg/m^2^	194	Transverse, Pfannenstiel except one subumbilical	Vertical, paramedian or midline above or below the umbilicus	WC; wound disruption or wound cellulitis	[26]
Brocato *et al.* (2013), USA	Retrospective cohort	2007–2011	All women underwent CD BMI >40 kg/m^2^	133	Transverse, Pfannenstiel	Vertical, supraumbilical	WC; superficial and deep surgical site infection, wound dehiscence or requiring take back to the operating room	[23]
Walton *et al*. (2017), USA	Retrospective cohort	2010–2015	Primary or repeated CD >23 weeks GA BMI ≥40 kg/m^2^	128	High transverse	Low transverse	WC; wound seroma, hematoma, dehiscence, requiring take back to the operating room, reopening, debriding or vacuuming the incision	[29]
Marrs *et al.*(2019), USA	RCT	2013–2017	Primary or repeated CD GA ≥24 weeks BMI ≥40 kg/m^2^	91	Transverse, Pfannenstiel	Vertical, midline, sub or suprapannicular	WC; surgical site infection, cellulitis, seroma/hematoma or separation up to 6 weeks postpartum	[27]
Dias *et al.* (2019), UK	Retrospective cohort	2010–2015	Primary or repeated CD GA >37 weeks BMI >40 kg/m^2^	453	Transverse, infra-panniculus; suprapubic Pfannenstiel or modified Cohen’s	Transverse, supra-panniculus; above the pannus	Wound infection; purulent drainage with/without laboratory confirmation, pain, tenderness, redness or heat	[28]
Thornburg *et al*. (2012), USA	Retrospective cohort	1994–2008	Primary CD BMI was ≥35 kg/m^2^	623	Low transverse, subpannicular	Vertical	WC; wound separation, spontaneous or indicated as result of seroma or wound infection/cellulitis	[24]
McLean *et al.* (2011), USA	Retrospective cohort	1998–2005	CD with BMI ≥30 kg/m^2^	238	Transverse	Vertical	Wound separation; partial or complete wound separation	[44]
Wall *et al.* (2003), USA	Retrospective cohort	1994–2000	Primary CD BMI was ≥35 kg/m^2^	239	Transverse	Vertical	WC; cellulitis, purulent wound discharge, seroma or hematoma or any need to open the incision	[25]

CD: Cesarean delivery; GA: Gestational age; RCT: Randomized controlled trial; WC: Wound complication.

Among the included articles, eight studies discussed the effect of transverse skin incision and vertical skin incision on WCs. Walton *et al.* compared WCs between low and high transverse skin incision types [29]. Dias *et al.* addressed infra-panniculus versus supra-panniculus incisions [28]. BMI was relatively increased among patients who had a vertical incision compared with those with a transverse incision. The incidence of diabetes, hypertension and preeclampsia was also higher among patients with a vertical incision, except for the reported findings by Marrs *et al.* (48% diabetic patients in transverse vs 32% in the vertical group). Sutton *et al.*, Bell *et al.* and Brocato *et al.* [21–23] reported that the rates of classical hysterotomy are significantly higher in pregnant patients with a vertical incision compared with a transverse incision, while low transverse uterine incisions were more frequent in transverse incision groups. Other patients’ clinical characteristics and variables are provided in Supplementary Appendix 3.

**Figure 2.**
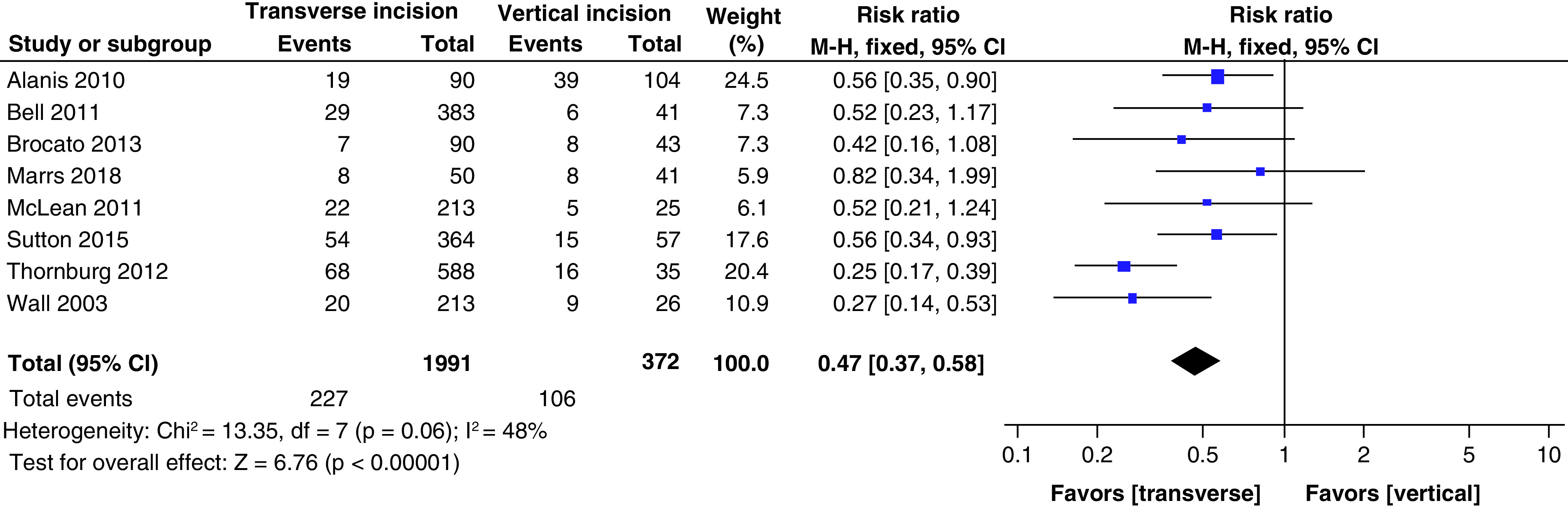
Forest plot of risk ratios for wound complication events.

WCs were more common in pregnant patients with vertical skin incisions when compared with the transverse skin incisions, according to four studies that demonstrated a significant difference [21,24,25]. However, after adjusting for confounders, only Thornburg *et al.* found a statistically significant difference in WC rates between the two types of incisions [24]. The incidence of WCs in vertical incision groups varied from 14.6 to 55.8%, higher than the transverse incision groups, where the incidence varied from 7.6 to 21.1%. Our pooled analysis of eight studies showed that the transverse incision was associated with a reduced risk of developing WCs from CD (relative risk [RR] = 0.47, 95% CI: 0.37–0.58; p < 0.00001) when compared with vertical incision (Figure 2).

Five studies reported that vertical type incisions are associated with higher estimated blood loss to compare with transverse type incision [22,23,25–28]; however, only three of those have reached statistically significant differences. Similarly, five studies stated that the transfusion rates were higher in the vertical group when compared with transverse group incision [21–23,26,27]; however, none of the respective studies reached statistical significance after adjusted comparison.

The length of hospital stay after delivery was reported in seven studies, with no difference between the vertical and transverse incision groups [21–23,25,27–29]. Total operative time was longer in those with a vertical skin incision; only three of the five studies reporting this notion demonstrated a statistically significant difference (Table 2) [22,23,28]. Interestingly the Dias *et al.* study reported very prolonged operative times compared with all of the included studies (97 ± 31 vs 143 ± 41) [28]. This study was performed in two maternity hospitals of Scotland, in women with very severe obesity (BMI ≥40 kg/m^2^); this is in contrast with the rest of the studies, which have been performed in the USA. The results of this study may also represent slightly different CD techniques used in the United Kingdom. However, prolonged operative time did not increase the WC rate in either group for the Dias *et al.* study (22.2 vs 27.7%) [28].

**Table 2.** Outcomes of included studies.

Author (year)	Patients (n)	Wound complication			Blood loss (ml)		Transfusion			Length of stay (day)		Operative time (min)		Ref.
		n (%)		aOR (95% CI)			n (%)		aOR (95% CI)					
Sutton *et al.* (2015)	364 in Subpannicular transverse	54 (14.8)	p = 0.03	1.7 (0.7–4.1)	900 (800–1000)	p < 0.001	7 (1.9)	p = 0.048	4.2 (0.9–19.0)	4 (3–4)	p = 0.6	55 (44–65)	p < 0.001	[22]
	57 in Vertical	15 (26.3)			1000 (900–1000)		4 (7.0)			4 (3–4)		73 (58–107)		
Bell *et al*. (2011)	383 in Low transverse	29 (7.6)	p = 0.03	1.91 (0.57–6.44)	NA		6 (1.6)	p = 0.01	2.78 (0.42–18.40)	4 (2–25)	p = 0.02	NA		[21]
	41 in Vertical	6 (14.6)					4 (9.8)			4 (2–45)				
Alanis *et al.* (2010)	90 in Pfannenstiel	19 (21.1)	NA	NA	23 (25.6)^†^	p = 0.007	9 (10)	p = 0.92	NA	NA		58.5 (49.0–73.0)	p < 0.001	[26]
	104 in Vertical	39 (37.5)			46 (44.2)^†^		10 (9.6)					71.0 (61–90.5)		
Brocato *et al.* (2013)	90 in Pfannenstiel	7 (8)	p = 0.08	NA	11 (12)^†^	p = 0.005	5 (6)	p = 0.056	NA	59 (66)^‡^	p = 0.1912	68 ± 30	p < 0.0001	[23]
	43 in Supraumbilical vertical	8 (19)			14 (33)^†^		7 (16)			33 (77)^‡^		97 ± 38		
Walton *et al.* (2017)	32 in High transverse	5 (15.63)	p = 0.24	NA	NA		NA			3.0 (2.0–4.0)	p = 0.13	NA		[29]
	96 in Low transverse	26 (27.08)								2.0 (2.0–3.0)				
Marrs *et al.* (2019)	50 in Pfannenstiel	8 (18.6)	p = 0.71	NA	800 (700–1000)	p = 0.13	4 (8)	p = 0.87	NA	3.2 ± 1	p = 0.40	68 ± 32	p = 0.57	[27]
	41 in Vertical	8 (21.1)			1000 (800–1000)		3 (7)			3.5 ± 4.1		69 ± 21		
Dias *et al.* (2019)	406 in Infra-panniculus transverse	90 (22.2)	p = 0.40	NA	705.1 ± 424.5	p = 0.004	NA			3.4 ± 2.4	p = 0.02	97 ± 31	p < 0.001	[28]
	47 in Supra-panniculus transverse	13 (27.7)			1044.5 ± 744.8					3.9 ± 1.8		143 ± 41		
Thornburg *et al.* (2012)	588 in Low transverse	68 (11.6)	p < 0.001	6.5 (3.2–13.4)	NA		NA			NA		NA		[24]
	35 in Vertical	16 (45.7)												
McLean *et al.* (2011)	213 in Transverse	22 (10)	p = 0.15	NA	NA		NA			NA		NA		[44]
	25 in Vertical	5 (20)												
Wall *et al.* (2003)	213 in Transverse	20 (9.4)	p = 0.001	NA	909 ± 316.7	p = 0.92	NA			4.4 ± 2.2	p = 0.16	68 ± 22.0	p = 0.16	[25]
	26 in Vertical	9 (34.6)			915 ± 249.3					5.0 ± 1.3		74 ± 19.2		

Data are in n (%), mean ± SD and median (1st–3rd quartile).

† Blood loss >1000 ml, n (%).

‡ Length of stay >3 days, n (%).

aOR: Adjusted odds ratio; NA: Not available; SD: Standard deviation.

**Table 3.** Clinical characteristics of study population for each included study.

Author (year)	Patients (n)	Age (year)	Gestational age (week)	BMI (kg/m^2^)	Chronic HTN	Diabetes	Tobacco use	Preeclampsia	Antibiotics prophylaxis	Tubal ligation	Classical hysterotomy		Ref.
Sutton *et al.* (2015)	364 in Subpannicular transverse	28.1 ± 6.3	38.7 ± 1.5	47.6 ± 6.2	101 (27.8)	104 (28.7)	NA	NA	NA	NA	28 (7.7)	p < 0.001, aOR 4.8 (95% CI: 2.2–10.4)	[22]
	57 in Vertical	31.4 ± 5.4	38.0 ± 1.5	54.5 ± 12.1	26 (45.6)	35 (62.5)					25 (43.9)		
Bell *et al.* (2011)	383 in Low transverse	26.7 ± 5.8	37.7 ± 3.3	41.7 ± 6.7	NA	NA	NA	NA	NA	74 (19.3)	28 (7.3)	p < 0.001	[21]
	41 in Vertical	31.0 ± 6.2	36.9 ± 3.4	48.2 ± 9.1						14 (34.1)	27 (65.9)		
Alanis *et al.* (2010)	90 in Pfannenstiel	28.0 (24–33)	39.0 (36–39)	52.8 (51.1–57.1)	37 (41.1)	17 (18.9)	11 (12.2)	20 (22.2)	65 (72.2)	NA	8 (8.9)	p < 0.001	[26]
	104 in Vertical	31.0 (26–34)	38.0 (36–39)	56.1 (51.9–59.8)	47 (45.2)	41 (39.4)	11 (10.6)	31 (29.8)	58 (55.8)		44 (42.3)		
Brocato *et al.* (2013)	90 in Pfannenstiel	26 ± 6	38.3 ± 3	56 ± 6	39 (43)	8 (9)	10 (11)	31 (34)	90 (100)	NA	7 (8)	p < 0.0001, OR: 24.6 (95% CI: 9–66.0)	[23]
	43 in Supraumbilical vertical	32 ± 5	36.5 ± 2.9	64 ± 10	23 (53)	21 (49)	4 (9)	19 (44)	43 (100)		29 (67)		
Walton *et al.* (2017)	32 in High transverse	28.8 ± 4.3	36.8 ± 2.9	49.9 ± 9.0	18 (56.25)	15 (46.88)	7 (21.88)	NA	NA	NA	NA	NA	[29]
	96 in Low transverse	29.2 ± 5.5	38.0 ± 1.9	49.8 ± 7.6	35 (36.46)	45 (46.88)	21 (21.88)						
Marrs *et al.* (2018)	50 in Pfannenstiel	30 ± 7	37 ± 2.2	50 ± 8	NA	24 (48)	3 (6)	NA	49 (98)	17 (34)	NA	NA	[27]
	41 in Vertical	28 ± 7	36.9 ± 3.5	48 ± 6		13 (32)	1 (2)		41 (100)	12 (29)			
Dias *et al.* (2019)	406 in Infra-panniculus transverse	30.6 ± 5.7	39.3 ± 2.07	43.3 ± 3.3	NA	89 (21.9)	68 (16.7)	NA	NA	NA	NA	NA	[28]
	47 in Supra-panniculus transverse	32.9 ± 4.4	38.2 ± 2.1	49.2 ± 7.1		20 (42.6)	4 (8.5)				0/47		
Thornburg *et al.* (2012)	588 in Low transverse	29.0 ± 6.0	37.3 ± 3.8	39.4 ± 6.7	NA	106 (17)	NA	27 (4.3)	623 (100)	NA	NA		[24]
	35 in Vertical												
McLean *et al.* (2011)	213 in Transverse	29.9 ± 6.0	39 (37–39)	36 (32–40)	55 (26)	49 (23)	44 (21)	28 (13)	NA	NA	NA		[44]
	25 in Vertical	29.4 ± 5.2	39 (37–39)	43 (36–51)	11 (44)	13 (52)	8 (32)	7 (28)					
Wall *et al.* (2003)	213 in Transverse	28.9 ± 6.2	37.6 ± 3.9	41.2 ± 4.8	NA	40 (18.8)	38 (18)	44 (20.7)	187 (87.8)	NA	NA		[25]
	26 in Vertical	27.5 ± 6.2	36.2 ± 5.2	44.1 ± 6.0		5 (19.2)	6 (23)	9 (34.6)	24 (92.3)				

aOR: Adjusted odds ratio; HTN: Hypertension; NA: Not available; OR: Odds ratio.

There was a trend that high transverse and suprapannicular incisions are associated with a lower risk of WCs compared with low transverse and infrapannicular incisions described in Walton *et al.* and Dias *et al.*, respectively [28,29]; however, there was no statistically significant difference between the groups.

## Discussion

Our main finding was that there is a lower risk of WCs in obese pregnant patients undergoing CD with a transverse skin incision compared with a vertical skin incision (RR = 0.47, 95% CI: 0.37–0.58; p < 0.00001). The results of our study coincide with findings described by Mccurdy *et al.* where vertical incisions were associated with a RR of 2.07 (95% CI: 1.61–2.67) for WCs compared with transverse incisions; however, significant possible confounders were present [30].

Multiple techniques have been studied to diminish the risk of infectious complications among pregnant patients undergoing CD, including adjunctive intravenous azithromycin to standard antibiotic prophylaxis for c-section in labor or after membrane rupture [31], vaginal preparation with povidone-iodine before cesarean section and applying negative-pressure wound dressing [32]. However, neither adjunction of azithromycin nor vaginal cleansing was investigated regarding prevention infections complications in obese parturients. Application of a negative wound dressing technique demonstrated no benefit in preventing surgical site infection in the obese population [32].

Other investigators addressed the influence of surgical technique on the risk of CD surgical site infection. Low transverse type of skin incision, or Pfannenstiel, or infrapannicular in obese parturient performed under pannus irrespective of the distance from the pubic bone poses significant technical difficulties and remains an issue of debate due to the presence of the subpannicular skin fold. In light of the above, a midline vertical skin incision is more frequently suggested for obese patients [21,33]. This is despite the higher risk of postoperative pain, superficial and fascial wound dehiscence, and postoperative atelectasis associated with midline vertical skin incisions [25].

High transverse skin incision theoretically reduces WCs because a higher abdominal wall incision avoids the need to incise through the pannus and has the added advantage of transverse incision such as good tensile strength. High transverse skin incision in obese parturient or suprapannicular, describes transverse incision performed above the pannus or suprapannicular. Since pannus retraction is not necessary, less thoracic compression and resultant hypotension are noted. High transverse skin incisions are also associated with reduced postoperative pain, lower rates of dehiscence and the possible risk of infection or bleeding [29]. Despite the aforementioned research, insufficient definitive guidance is available to assist obstetricians in the selection of the best incision type to use for obese patients undergoing CD.

Given the observed increase in the risk of WCs with the use of vertical skin incisions, physicians and researchers must attempt to identify further interventions and modifications to the commonly used incisions, including innovative surgical techniques, which can reduce the overall postoperative morbidity in CD [14].

In a national anonymous survey of obstetrics and gynecology (OB/GYN) physicians, when Smid *et al.* assessed the preferences of obstetricians regarding cesarean incision practices in morbidly obese pregnant patients, the responders stated a preference for Pfannenstiel incision with a taping of the pannus [34]. Another multicenter anonymous survey regarding women’s preference and concerns for cesarean skin incision among pregnant with class III obesity showed that patients prioritize immediate maternal and fetal safety over other concerns including cosmetic outcome [35].

There is potential for reduction of post-CD WCs through surgical techniques such as high transverse incision and suprapannicular incisions described in Walton *et al.* and Dias *et al.*, respectively [28,29]. Both authors Walton *et al.* and Dias *et al.* observed a trend showing a reduction of WC with high transverse and suprapannicular type of skin incision when compared with low transverse and infrapannicular, respectively [28,29]; however, the difference did not reach statistical significance. These data demonstrate the necessity for further investigation of best skin incision techniques in obese parturients.

The rate of WCs increases with an increase of BMI from 9.2% in women with BMI 30–39.9 kg/m^2^ (aOR: 1.4, 95% CI: 0.99–2.0; p = 0.06), to 16.8% in women with BMI 40–49.9 kg/m^2^ (aOR: 2.6, 95% CI: 1.7–3.8; p < 0.01). The rate of WC in BMI >50 kg/m^2^ is 22.9% (aOR: 3.0, 95% CI: 1.9–4.9; p < 0.01) [36]. Potentially confounding any investigation of postoperative WCs are the medical comorbidities these patients often exhibit. Minor or unreported differences in operative technique or preparation also play a role in the risk for the development of postoperative WCs. Factors such as smoking, chronic medical conditions such as diabetes and hypertension [37], perioperative antibiotic use [31], pre-operative skin preparation [38], tissue closure [39], wound vacuum [32], and the use of drains [40–43], all increase the risk of postoperative wound infection. Influence of comorbidity and technique confounders required analysis of surgical incision choice and the likelihood of postoperative surgical complications.

Our review’s primary strength is that our investigation is a robust systematic review to compare different cesarean skin incision types and evaluate their postoperative effects among obese pregnant patients. Our systematic review utilized several databases to ensure proper coverage of the topic. The search strategy was maximally simplified and transparent, which makes it easily replicable. Additionally, we strictly followed the inclusion criteria that allowed us to focus on the research topic and include only those articles that describe the association between the type of skin incision and WCs in the obese obstetric population. Two independent reviewers performed screening of abstracts. We included only articles available in full text. All of these approaches provide reliable estimates about the effects of incision type on WCs in obese obstetric patients.

Our analysis has several inherent limitations because of the limited available literature addressing this topic. Nine of the ten studies which met the inclusion criteria were retrospective cohort studies. Only one RCT met the inclusion criteria. The predominance of retrospective cohort studies used lowers the level of evidence for this review. We were unable to compute pooled estimates for the cohort studies without access to individual patients’ data. The specific incision technique and outcome definitions were heterogeneous and varied somewhat among the different studies. A half of the reviewed studies has not reported whether prophylactics antibiotics were utilized, which makes generalizability of results limited. Finally, a significant limitation of any retrospective cohort drove review of literature is in type of patient selection. Decision strategies insofar as the choice of the incision are a limitation. Nonetheless, our review is important because its analysis permits the development of a steppingstone for a robust understanding of this topic.

## Conclusion

In conclusion, transverse skin incision may be preferable to vertical skin incision in pregnant patients with obesity as it may be associated with lower rates of WCs. Further scientific evaluation of our conclusions and correlations necessitates a prospective randomized trial.

## Future perspective

Our vision is that more RCT studies will be performed to describe the best skin incision type for cesarean section in obese patients. Also, more RCTs investigating different types of transverse skin incisions will appear, allowing us to perform systematic reviews and meta-analysis to describe the association between supra and infrapinnacular incision type and WCs in obese patients with CD.

**Executive summary**

- Insufficient definitive guidance is available to assist obstetricians in the selection of the best incision type to use for obese pregnant patients undergoing cesarean delivery (CD).
- Our systematic review of the literature is consistent with other publications in that transverse skin incision may be preferable to vertical skin incision at CD in pregnant patients with obesity as it may be associated with a lower rate of wound complications.
- Further investigation of best skin incision techniques in obese parturients during CD is still needed.

## References

[1] Olyaeemanesh A, Bavandpour E, Mobinizadeh M, Ashrafinia M, Bavandpour M, Nouhi M Comparison of the Joel-Cohen-based technique and the transverse Pfannenstiel for cesarean section for safety and effectiveness: a systematic review and meta-analysis. Med. J. Islam. Repub. Iran 31, 54 (2017). 29445683 10.14196/mjiri.31.54 PMC5804473

[2] Poobalan AS, Aucott LS, Gurung T, Smith WC, Bhattacharya S Obesity as an independent risk factor for elective and emergency cesarean delivery in nulliparous women – systematic review and meta-analysis of cohort studies. Obes. Rev. 10(1), 28–35 (2009). 19021871 10.1111/j.1467-789X.2008.00537.x

[3] Vitale SG, Marilli I, Cignini P Comparison between modified Misgav-Ladach and Pfannenstiel-Kerr techniques for cesarean section: review of literature. J. Prenat. Med. 8(3–4), 36–41 (2014). 26265999 PMC4510561

[4] Anderson LN, Knight JA, Hung RJ The Ontario Birth study: a prospective pregnancy cohort study integrating perinatal research into clinical care. Paediatr. Perinat. Epidemiol. 32(3), 290–301 (2018). 29750375 10.1111/ppe.12473

[5] Flegal KM, Kruszon-Moran D, Carroll MD, Fryar CD, Ogden CL Trends in obesity among adults in the United States, 2005 to 2014. JAMA 315(21), 2284–2291 (2016). 27272580 10.1001/jama.2016.6458 PMC11197437

[6] Baeten JM, Bukusi EA, Lambe M Pregnancy complications and outcomes among overweight and obese nulliparous women. Am. J. Public Health 91(3), 436–440 (2001). 11236410 10.2105/ajph.91.3.436 PMC1446581

[7] Hibbard JU, Gilbert S, Landon MB National Institute of Child Health and Human Development Maternal-Fetal Medicine Units Network. Trial of labor or repeat cesarean delivery in women with morbid obesity and previous cesarean delivery. Obstet. Gynecol. 108(1), 125–133 (2006). 16816066 10.1097/01.AOG.0000223871.69852.31

[8] Cedergren MI Maternal morbid obesity and the risk of adverse pregnancy outcome. Obstet. Gynecol. 103(2), 219–224 (2004). 14754687 10.1097/01.AOG.0000107291.46159.00

[9] Figueroa D, Jauk VC, Szychowski JM Surgical staples compared with subcuticular suture for skin closure after cesarean delivery: a randomized controlled trial. Obstet. Gynecol. 121, 33–38 (2013). 23262925 10.1097/aog.0b013e31827a072c PMC3875219

[10] Sebire N, Jolly M, Harris J, Wadsworth J, Joffe M, Beard RW Maternal obesity and pregnancy outcome: a study of 287,213 pregnancies in London. Int. J. Obes. Relat. Metab. Disord. 25, 1175–1182 (2001). 11477502 10.1038/sj.ijo.0801670

[11] Jensen DM, Damm P, Sørensen B Pregnancy outcome and prepregnancy body mass index in 2459 glucose-tolerant Danish women. Am. J. Obstet. Gynecol. 189(1), 239–244 (2003). 12861169 10.1067/mob.2003.441

[12] Weiss JL, Malone FD, Emig D Obesity, obstetric complications and cesarean delivery rate – a population-based screening study. Am. J. Obstet. Gynecol. 190(4), 1091–1097 (2004). 15118648 10.1016/j.ajog.2003.09.058

[13] Perlow JH, Morgan MA Massive maternal obesity and perioperative cesarean morbidity. Am. J. Obstet. Gynecol. 170(2), 560–565 (1994). 8116713 10.1016/s0002-9378(94)70227-6

[14] Conner SN, Verticchio JC, Tuuli MG, Odibo AO, Macones GA, Cahill AG Maternal obesity and risk of postcesarean wound complications. Am. J. Perinatol. 31(4), 299–304 (2014). 23765707 10.1055/s-0033-1348402 PMC3796045

[15] Robinson HE, O'Connell CM, Joseph KS, McLeod NL Maternal outcomes in pregnancies complicated by obesity. Obstet. Gynecol. 106(6), 1357–1364 (2005). 16319263 10.1097/01.AOG.0000188387.88032.41

[16] Higgins J, Green S The Cochrane Collaboration. Cochrane handbook for systematic reviews of interventions version 5.1.0 [updated March 2011]. (2011). http://handbook.cochrane.org

[17] Moher D, Liberati A, Tetzlaff J, Altman DG Group TP. Preferred reporting items for systematic reviews and meta-analyses: the PRISMA statement. PLoS Med. 6(7), e1000097 (2009). 10.1371/journal.pmed.1000097 19621072 PMC2707599

[18] Wells G, Shea B, O'Connell J The Newcastle-Ottawa scale (NOS) for assessing the quality of nonrandomised studies in meta-analyses. (2014). http://med.mercer.edu

[19] Higgins JP, Altman DG, Gøtzsche PC The Cochrane Collaboration’s tool for assessing risk of bias in randomized trials. BMJ 18, 343 (2011). 10.1136/bmj.d5928 PMC3196245 22008217

[20] Egger M, Davey Smith G, Schneider M, Minder C Bias in meta-analysis detected by a simple, graphical test. BMJ 13, 315 (1997). 10.1136/bmj.315.7109.629 PMC2127453 9310563

[21] Bell J, Bell S, Vahratian A, Awonuga AO Abdominal surgical incisions and peri-operative morbidity among morbidly obese women undergoing cesarean delivery. Eur. J. Obstet. Gynecol. Reprod. Biol. 154(1), 16–19 (2011). 20832161 10.1016/j.ejogrb.2010.07.043

[22] Sutton AL, Sanders LB, Subramaniam A, Jauk VC, Edwards RK Abdominal incision selection for cesarean delivery of women with class III obesity. Am. J. Perinatol. 33(6), 547–551 (2016). 26692204 10.1055/s-0035-1570339

[23] Brocato BE, Thorpe EM, Gomez LM, Wan JY, Mari G The effect of cesarean delivery skin incision approach in morbidly obese women on the rate of classical hysterotomy. J. Pregnancy. 890296 (2013). 24349784 10.1155/2013/890296 PMC3853441

[24] Thornburg LL, Linder MA, Durie DE, Walker B, Pressman EK, Glantz JC Risk factors for wound complications in morbidly obese women undergoing primary cesarean delivery. J. Matern. Fetal Neonatal Med. 25(9), 1544–1548 (2012). 22233403 10.3109/14767058.2011.653422

[25] Wall PD, Deucy EE, Glantz JC, Pressman EK Vertical skin incisions and wound complications in the obese parturient. Obstet. Gynecol. 102(5, Part 1), 952–956 (2003). 14672469 10.1016/s0029-7844(03)00861-5

[26] Alanis MC, Villers MS, Law TL, Steadman EM, Robinson CJ Complications of cesarean delivery in the massively obese parturient. Am. J. Obstet. Gynecol. 203(3), 271 (2010). 20678746 10.1016/j.ajog.2010.06.049

[27] Marrs C, Blackwell S, Hester A Pfannenstiel versus vertical skin incision for cesarean delivery in women with class III obesity: a randomized trial. Am. J. Perinatol. 36(1), 97–104 (2019). 30060292 10.1055/s-0038-1667287

[28] Dias M, Dick A, Reynolds RM, Lahti-Pulkkinen M, Denison FC Predictors of surgical site skin infection and clinical outcome at cesarean section in the very severely obese: a retrospective cohort study. PLoS ONE 14(6), e0216157 (2019). 31246973 10.1371/journal.pone.0216157 PMC6598740

[29] Walton R, Shnaekel K, Ounpraseuth S, Napolitano P, Magann E High transverse skin incisions may reduce wound complications in obese women having cesarean sections: a pilot study. J. Matern. Fetal Neonatal Med. 32, 1–11 (2017). 29020834 10.1080/14767058.2017.1391780

[30] Mccurdy RJ, Felder LA, Saccone G The association of skin incision placement during cesarean delivery with wound complications in obese women: a systematic review and meta-analysis. J. Matern. Fetal Neonatal Med. 1, 13 (2020). 10.1080/14767058.2020.1786050 32631122

[31] Bratzler DW, Dellinger EP, Olsen KM Clinical practice guidelines for antimicrobial prophylaxis in surgery. Am. J. Health Syst. Pharm. 70(3), 195–283 (2013). 23327981 10.2146/ajhp120568

[32] Swift SH, Zimmerman MB, Hardy-Fairbanks AJ Effect of single-use negative pressure wound therapy on postcesarean infections and wound complications for high-risk patients. J. Reprod. Med. 60(5–6), 211–218 (2015). 26126306

[33] Gallup DG Modifications of cellotomy techniques to decrease morbidity in obese gynecologic patients. Am. J. Obstet. Gynecol. 150(2), 171–178 (1984). 6383048 10.1016/s0002-9378(84)80012-5

[34] Smid MC, Smiley SG, Schulkin J, Stamilio DM, Edwards RK, Stuebe AM The problem of the pannus: physician preference survey and a review of the literature on cesarean skin incision in morbidly obese women. Am. J. Perinatol. 33(05), 463–472 (2016). 26510932 10.1055/s-0035-1566000

[35] Smid MC, Edwards RK Class III obese women’s preferences and concerns for cesarean skin incision: a multicenter survey. Am. J. Perinatol. 34(3), 289–294 (2017). 27533104 10.1055/s-0036-1586750

[36] Conner SN, Verticchio JC, Tuuli MG, Odibo AO, Macones GA, Cahill AG Maternal obesity and risk of postcesarean wound complications. Am. J. Perinatol. 31(4), 299–304 (2014). 23765707 10.1055/s-0033-1348402 PMC3796045

[37] Palatnik A, Grobman WA The association of skin-incision type at cesarean with maternal and neonatal morbidity for women with multiple prior cesarean deliveries. Eur. J. Obstet. Gynecol. Reprod. Biol. 191, 121–124 (2015). 26117440 10.1016/j.ejogrb.2015.06.009

[38] Tuuli MG, Liu J, Stout MJ A randomized trial comparing skin antiseptic agents at cesarean delivery. N. Engl. J. Med. 374(7), 647–655 (2016). 26844840 10.1056/NEJMoa1511048 PMC4777327

[39] Tuuli MG, Stout MJ, Martin S, Rampersad RM, Cahill AG, Macones GA Comparison of suture materials for subcuticular skin closure at cesarean delivery. Am. J. Obstet. Gynecol. 215(4), 490 (2016). 27179440 10.1016/j.ajog.2016.05.012

[40] Anderson R, Gates S, Anderson ER Techniques and materials for closure of the abdominal wall in cesarean section. Cochrane Database Syst. Rev. (4), 2003 10.1002/14651858.CD004663 PMC9036625 15495122

[41] Al-Inany H, Youssef G, ElMaguid A, Hamid M, Hosni A Value of subcutaneous drainage system in obese females undergoing cesarean section using Pfannenstiel incision. Gynecol. Obstet. Invest. 53, 75–78 (2002). 11961377 10.1159/000052996

[42] Magann EF, Chauhan SP, Rodts-Palenik S, Bufkin L, Martin JN Jr, Morrison JC Subcutaneous stitch closure versus subcutaneous drain to prevent wound disruption after cesarean delivery: a randomized clinical trial. Am. J. Obstet. Gynecol. 186(6), 1119–1123 (2002). 12066083 10.1067/mob.2002.123823

[43] Ramsey PS, White AM, Guinn DA Subcutaneous tissue reapproximation, alone or in combination with drain, in obese women undergoing cesarean delivery. Obstet. Gynecol. 105, 967–973 (2005). 15863532 10.1097/01.AOG.0000158866.68311.d1

[44] McLean M, Hines R, Polinkovsky M Type of skin incision and wound complications in the obese parturient.. Am J Perinatol. 29(4), 301–306 (2012). 22105439 10.1055/s-0031-1295637

