# Supplementary material for: Maternal morbidity associated with skin incision type at cesarean delivery in obese patients: a systematic review
Source: Future Sci OA. 2020 Dec 18;7(3):FSO669. doi: 10.2144/fsoa-2020-0160 (PMC7850001; doi:10.2144/fsoa-2020-0160)
Supplement: Supplementary file 1 [file fsoa-07-669-s1.docx]

**Appendix S1**: Detailed search strategy for each database.

| **Database** | **Search Strategy** | **Results** |
| --- | --- | --- |
| **PubMed** | (Cesarean OR (Cesarean section) OR (caesarean section) OR CS OR (cesarean delivery) OR (C-Section) OR (C Section) OR (Postcesarean Section) OR (post-cesarean delivery)) AND ((abdominal incision) OR (Skin incision) OR (Vertical skin incision) OR Pfannenstiel OR (transverse skin incision) OR (under umbilicus) OR (midline incision) OR (Suprapannicular incision)) AND (obesity OR obese OR (body mass index) OR (body weight) OR overweight) | 140 |
| **OVID** | 1 (Cesarean OR (Cesarean section) OR (caesarean section) OR CS OR (cesarean delivery) OR (C-Section) OR (C Section) OR (Postcesarean Section) OR (post-cesarean delivery)). ti,ab,kw. | 1 and 2 =19 |
|  | 2 ((abdominal incision) OR (Skin incision) OR (Vertical skin incision) OR Pfannenstiel OR (transverse skin incision) OR (under umbilicus) OR (midline incision) OR (Suprapannicular incision)). ti,ab,kw. | 2 and 3 = 10 |
|  | 3 (obesity OR obese OR (body mass index) OR (body weight) OR overweight). ti,ab,kw. | 1 and 3 = 210 |
| **Embase** | (Cesarean OR (Cesarean section) OR (caesarean section) OR CS OR (cesarean delivery) OR (C-Section) OR (C Section) OR (Postcesarean Section) OR (post-cesarean delivery)) AND ((abdominal incision) OR (Skin incision) OR (Vertical skin incision) OR Pfannenstiel OR (transverse skin incision) OR (under umbilicus) OR (midline incision) OR (Suprapannicular incision)) AND (obesity OR obese OR (body mass index) OR (body weight) OR overweight) | 343 |
| **SCOPUS** | TITLE-ABS-KEY ( Cesarean OR ( Cesarean section ) OR ( caesarean section ) OR CS OR ( cesarean delivery ) OR ( C-Section ) OR ( C Section ) OR ( Postcesarean Section ) OR ( post-cesarean delivery ) ) AND TITLE-ABS-KEY ( ( abdominal incision ) OR ( Skin incision ) OR ( Vertical skin incision ) OR Pfannenstiel OR ( transverse skin incision ) OR ( under umbilicus ) OR ( midline incision ) OR ( Suprapannicular incision ) ) AND TITLE-ABS-KEY ( obesity OR obese OR ( body mass index ) OR ( body weight ) OR overweight ) | 161 |
| **Web of Science** | (Cesarean OR (Cesarean section) OR (caesarean section) OR CS OR (cesarean delivery) OR (C-Section) OR (C Section) OR (Postcesarean Section) OR (post-cesarean delivery)) AND ((abdominal incision) OR (Skin incision) OR (Vertical skin incision) OR Pfannenstiel OR (transverse skin incision) OR (under umbilicus) OR (midline incision) OR (Suprapannicular incision)) AND (obesity OR obese OR (body mass index) OR (body weight) OR overweight) | 131 |
| **Cochrane Library** | (Cesarean OR (Cesarean section) OR (caesarean section) OR CS OR (cesarean delivery) OR (C-Section) OR (C Section) OR (Postcesarean Section) OR (post-cesarean delivery)) AND ((abdominal incision) OR (Skin incision) OR (Vertical skin incision) OR Pfannenstiel OR (transverse skin incision) OR (under umbilicus) OR (midline incision) OR (Suprapannicular incision)) AND (obesity OR obese OR (body mass index) OR (body weight) OR overweight) | 83 |
| **Clinicaltrial.gov** | Condition or Disease:  (Cesarean OR (Cesarean section) OR (caesarean section) OR CS OR (cesarean delivery) OR (C-Section) OR (C Section) OR (Postcesarean Section) OR (post-cesarean delivery)) Other Terms: ((abdominal incision) OR (Skin incision) OR (Vertical skin incision) OR Pfannenstiel OR (transverse skin incision) OR (under umbilicus) OR (midline incision) OR (Suprapannicular incision)) AND (obesity OR obese OR (body mass index) OR (body weight)) | 38 |

**Appendix S2a**: Quality assessment of cohort studies using a modified Newcastle-Ottawa scale

| Study ID | Selection | | | | Comparability | Outcome | | | Total (9) |
| --- | --- | --- | --- | --- | --- | --- | --- | --- | --- |
|  | Representativeness of exposed cohort (⋆) | Selection of non-exposed cohort (⋆) | Ascertainment of exposure (⋆) | Demonstration that outcome of interest was not present at start of study (⋆) | (⋆⋆) | Assessment of outcome (⋆) | Length of follow-up (⋆) | Adequacy of follow up (⋆) |  |
| Sutton, 2015 | ⋆ | ⋆ | ⋆ | ⋆ | ⋆⋆ | ⋆ | ⋆ | ⋆ | 9 |
| Bell, 2011 | ⋆ | ⋆ | ⋆ | ⋆ | ⋆⋆ | ⋆ | - | - | 7 |
| Alanis, 2010 | ⋆ | ⋆ | ⋆ | ⋆ | - | ⋆ | ⋆ | ⋆ | 7 |
| Brocato, 2013 | ⋆ | ⋆ | ⋆ | ⋆ | ⋆⋆ | ⋆ | ⋆ | ⋆ | 9 |
| Walton, 2017 | ⋆ | ⋆ | ⋆ | ⋆ | ⋆⋆ | ⋆ | ⋆ | ⋆ | 9 |
| Dias, 2019 | ⋆ | ⋆ | ⋆ | ⋆ | ⋆ | ⋆ | ⋆ | ⋆ | 8 |
| Thornburg, 2012 | ⋆ | ⋆ | ⋆ | ⋆ | ⋆ | ⋆ | ⋆ | ⋆ | 8 |
| McLean, 2011 | ⋆ | ⋆ | ⋆ | ⋆ | ⋆ | ⋆ | ⋆ | ⋆ | 8 |
| Wall, 2003 | ⋆ | ⋆ | ⋆ | ⋆ | - | ⋆ | ⋆ | ⋆ | 7 |

**Appendix 2b:** Authors' judgments and justifications of the included RCT using Cochrane assessment table for the risk of bias.

| **Reason/Quotation** | **Risk of bias** | **Marrs 2018** |
| --- | --- | --- |
| Randomization was computer-generated 1:1 randomization with stratification for site | Low risk | Random sequence generation (selection bias) |
| Randomization sequences were created for each site, and ordered opaque envelopes were created by the principal investigator. Randomization envelopes were kept in a secure location in the operating suite at each site. | Low risk | Allocation concealment (selection bias) |
| Masking of the physician or patient to the assignment was not feasible due to the nature of intervention. | Low | Blinding of participants and personnel (performance bias) |
|  | Low risk | Blinding of outcome assessment (detection bias) |
| No loss to follow-up. | Low risk | Incomplete outcome data (attrition bias) |
| All outcomes were reported. | Low risk | Selective reporting (reporting bias) |
|  | Unclear | Other bias |

| Author, year | Number of Patients | Age  (y) | Gestational age (wk) | BMI (kg/m^2^) | Chronic HTN | Diabetes | Tobacco Use | Preeclampsia | Antibiotics prophylaxis | Tubal ligation | Classical hysterotomy |
| --- | --- | --- | --- | --- | --- | --- | --- | --- | --- | --- | --- |
| Sutton, 2015 | 364 in Subpannicular Transverse | 28.1 ± 6.3 | 38.7 ± 1.5 | 47.6 ± 6.2 | 101 (27.8) | 104 (28.7) | NR | NR | NR | NR | 10 (2.8) |
|  | 57 in Vertical | 31.4 ± 5.4 | 38.0 ± 1.5 | 54.5 ± 12.1 | 26 (45.6) | 35 (62.5) |  |  |  |  | 21 (36.8) |
| Bell, 2011 | 383 in Low Transverse | 26.7 ± 5.8 | 37.7 ± 3.3 | 41.7 ± 6.7 | NR | NR | NR | NR | NR | 74 (19.3) | 28 (7.3) |
|  | 41 in Vertical | 31.0 ± 6.2 | 36.9 ± 3.4 | 48.2 ± 9.1 |  |  |  |  |  | 14 (34.1) | 27 (65.9) |
| Alanis, 2010 | 90 in Pfannenstiel | 28.0 (24–33) | 39.0 (36–39) | 52.8 (51.1–57.1) | 37 (41.1) | 17 (18.9) | 11 (12.2) | 20 (22.2) | 65 (72.2) | NR | NR |
|  | 104 in Vertical | 31.0 (26–34) | 38.0 (36–39) | 56.1 (51.9–59.8) | 47 (45.2) | 41 (39.4) | 11 (10.6) | 31 (29.8) | 58 (55.8) |  |  |
| Brocato, 2013 | 90 in Pfannenstiel | 26 ± 6 | 38.3 ± 3 | 56 ± 6 | 39 (43) | 8 (9) | 10 (11) | 31 (34) | 90 (100) | NR | 7 (8) |
|  | 43 in Supraumbilical Vertical | 32 ± 5 | 36.5 ± 2.9 | 64 ± 10 | 23 (53) | 21 (49) | 4 (9) | 19 (44) | 43 (100) |  | 29 (67) |
| Walton, 2017 | 32 in High Transverse | 28.8 ± 4.3 | 36.8 ± 2.9 | 49.9 ± 9.0 | 18 (56.25) | 15 (46.88) | 7 (21.88) | NR | NR | NR | NR |
|  | 96 in Low Transverse | 29.2 ± 5.5 | 38.0 ± 1.9 | 49.8 ± 7.6 | 35 (36.46) | 45 (46.88) | 21 (21.88) |  |  |  |  |
| Marrs, 2018 | 50 in Pfannenstiel | 30 ± 7 | 37 ± 2.2 | 50 ± 8 | NR | 24 (48) | 3 (6) | NR | 49 (98) | 17 (34) | NR |
|  | 41 in Vertical | 28 ± 7 | 36.9 ± 3.5 | 48 ± 6 |  | 13 (32) | 1 (2) |  | 41 (100) | 12 (29) |  |
| Dias, 2019 | 406 in Infra-panniculus Transverse | 30.6 ± 5.7 | 39.3 ± 2.07 | 43.3 ± 3.3 | NR | 89 (21.9) | 68 (16.7) | NR | NR | NR | NR |
|  | 47 in Supra-panniculus Transverse | 32.9 ± 4.4 | 38.2 ± 2.1 | 49.2 ± 7.1 |  | 20 (42.6) | 4 (8.5) |  |  |  |  |
| Thornburg, 2012 | 588 in Low Transverse | 29.0 ± 6.0 | 37.3 ± 3.8 | 39.4 ± 6.7 | NR | 106 (17) | NR | 27 (4.3) | 623 (100) | NR | NR |
|  | 35 in Vertical |  |  |  |  |  |  |  |  |  |  |
| McLean, 2011 | 213 in Transverse | 29.9 ± 6.0 | 39 (37–39) | 36 (32–40) | 55 (26) | 49 (23) | 44 (21) | 28 (13) | NR | NR | NR |
|  | 25 in Vertical | 29.4 ± 5.2 | 39 (37–39) | 43 (36–51) | 11 (44) | 13 (52) | 8 (32) | 7 (28) |  |  |  |
| Wall, 2003 | 213 in Transverse | 28.9 ± 6.2 | 37.6 ± 3.9 | 41.2 ± 4.8 | NR | 40 (18.8) | 38 (18) | 44 (20.7) | 187 (87.8) | NR | NR |
|  | 26 in Vertical | 27.5 ± 6.2 | 36.2 ± 5.2 | 44.1 ± 6.0 |  | 5 (19.2) | 6 (23) | 9 (34.6) | 24 (92.3) |  |  |

**Appendix S3**: Clinical characteristics of studies’ population

Data are presented as n (%), Mean ± SD, and Median (1^st^ quartile–3^rd^ quartile)

Abbreviations: y, year; wk, week; HTN, hypertension; NR, not reported.
